# Factors associated with long‐term mortality for stroke unit patients in Latvia

**DOI:** 10.1002/brb3.1152

**Published:** 2018-11-12

**Authors:** Illa Mihejeva, Anita Vētra, Guna Bērziņa

**Affiliations:** ^1^ Riga Stradiņš University Riga Latvia; ^2^ Riga East University Hospital Riga Latvia

**Keywords:** disability, mortality, neurological symptoms, risk factors, stroke

## Abstract

**Aim:**

The aim of this study was to evaluate how pre‐stroke risk factors, neurological symptoms, and the level of disability shortly after stroke are associated with poststroke mortality during a 7‐year period after stroke, for persons treated in a stroke unit.

**Methods:**

The data of 231 patients were included in the study. Patients who were treated in the stroke unit at the Riga East University Hospital between February 1, 2009, and July 20, 2009, were included in this study. Three stepwise Cox proportional hazard analyses were performed to analyze mortality in the 7 years following stroke. Pre‐stroke risk factors (type of stroke, arterial hypertension, diabetes mellitus, atrial fibrillation, smoking, alcohol abuse, obesity, recurrent stroke, age, gender), neurological symptoms (motor deficit, sensory disturbance, aphasia, poststroke urinary incontinence (PSUI), mental status), and limitations of activity (feeding, bathing, grooming, dressing, toilet use, transfers, mobility, stairs) were evaluated as factors associated with mortality after stroke.

**Results:**

A total of 145 (62.8%) patients died during the study period. The final model for each group of factors included only one of the factors used for the analysis. Patients who had alcohol abuse were 40% more likely to die earlier. The hazard for those with PSUI is 1.72 times higher than those without PSUI. The independence in grooming showed a 39% lower likelihood of dying earlier.

**Conclusion:**

Alcohol abuse as a pre‐stroke risk factor, poststroke urinary incontinence as a neurological symptom, and dependence in grooming as a factor of disability were associated with earlier mortality in the first seven years after stroke.

## 1 INTRODUCTION

Stroke remains one of the main causes of death and disability worldwide. Approximately six million persons die due to stroke annually (WHO, 2017). Reported stroke mortality in Latvia in 2014 was 119.5 per 100 000, which is 8.5% of all reported deaths and is the second leading cause of death behind ischemic heart disease (The Centre for Disease Prevention and Control of Latvia, 2015). According to the WHO, twice as many as die from stroke survive, most of the survivors live with disability. Therefore, this is a serious health problem leading to a burden on the individual, his/her family, and society (Feigin et al., 2014). Case‐fatality rates for ischemic stroke have declined by 26% on average across OECD (Organisation for Economic Co‐operation and Development) countries between 2000 and 2009. The trend is similar for hemorrhagic stroke with an average reduction of 17% during the same period. These reductions suggest widespread improvement in the organization and quality of care for stroke patients (OECD, 2011).

The organized inpatient care (stroke unit) is an approach to stroke patients that has shown superiority compared to other approaches (Nimptsch & Mansky, 2014). The core characteristics of stroke unit settings are multidisciplinary staffing (medical, nursing, and therapy staff—usually including physiotherapy, occupational therapy, speech therapy, and social work) and coordinated multidisciplinary team care incorporating meetings at least once a week (Sun, Paulus, Eyssen, Maervoet, & Saka, 2013).

Patients who had been treated in the stroke unit are more likely to survive, return home, and regain independence (Sun et al., 2013; Weder, 2015). Using the stroke unit model with interdisciplinary teams is more effective at treating stroke than aspirin/thrombolysis/statin use and lowering blood pressure (Langhorne, de Villiers, & Pandian, 2012). The most likely reasons for this superiority compared to other interventions include a more intensive approach to the management of medical complications and earlier and more focused rehabilitation (Sinha & Warburton, 2000).

The stroke units were introduced to the world during the 1950s. However, the first in Latvia was founded in 2000 in Riga, at “Gaiļezers” hospital. There has as yet been no information published on the outcomes from this particular stroke unit. Moreover, the exploration of important factors would be beneficial to implement more focused stroke care and therefore improve short‐ and long‐term outcomes, including survival.

Several risk factors have shown importance in affecting mortality after stroke, such as atrial fibrillation, diabetes, smoking, and previous stroke. The adequate control of these factors may significantly improve long‐term survival (Kammersgaard & Olsen, 2006). The severity of neurological deficits due to stroke, such as consciousness, and those included in the National Institute of Health Stroke Scale also have value in predicting mortality (Weimar, Ziegler, Konig, & Diener, 2002). Furthermore, the disability, functional improvements, and functional status at 6 months are a significant predictor of long‐term mortality after stroke (Scrutinio et al., 2015). Exploration of these important factors and their interaction would be beneficial for the implementation of more focused stroke care and therefore improvement of short‐ and long‐term outcomes, including survival.

The aim of this study was to evaluate how pre‐stroke risk factors, neurological symptoms, and disability shortly after stroke are associated with poststroke mortality in the 7 years after stroke for persons who have been treated in a stroke unit.

## 2 MATERIALS AND METHODS

Design of the study is single‐center prospective cohort study with consecutive sampling. The study population consisted of 251 patient who were treated at the stroke unit in Riga East University Hospital between February 1 and July 30 in 2009, with a diagnosis of stroke according to WHO criteria (Stroke, 1989) (I63, I60, I61, and I64, according to the 10th revision of the International Statistical Classification of Diseases and Related Health Problems). Patients who have been urgently hospitalized to the emergency department with suspected stroke are screened using computed tomography. If diagnosis of stroke is confirmed and no neurosurgery is needed, patients are transferred from emergency department to the stroke unit, where neurologist, nursing staff, physiotherapists, occupational therapists, and speech and language therapists provide acute stroke care on site in stroke unit and Physical and rehabilitation medicine doctor serves as consultant.

Data on risk factors of stroke and neurologic symptoms after stroke were gathered from patients’ medical records, as well as from evaluations of patients when they were undergoing treatment in the stroke unit. The assessment of patients was performed at day 7 after admission to hospital. Information about a person's death was gathered from the information system of the hospital, by follow‐up phone call to the patient or his/her relatives, between August and December 2015. The study conforms with the ethical principles of the Declaration of Helsinki and is approved by the Ethics Committee of Riga Stradiņš University, Latvia. Each participant or his/her next‐of‐kin signed informed consent for participation in the study.

Mortality was used as an outcome variable during the 7 years following stroke. (Patients without a date of death were censored at the last day of year last known alive.) As independent variables, pre‐stroke risk factors, neurological symptoms, and limitations of activities after stroke were used. Pre‐stroke risk factors that were used in the study were arterial hypertension, diabetes mellitus, atrial fibrillation, smoking, alcohol abuse, obesity, recurrent stroke, age (years), and gender. All these outcomes, except age, were reported in a dichotomized way (“0”—no; “1”—yes). Neurological symptoms following stroke were reported based on the NIH Stroke Scale. These symptoms were defined as motor deficit, sensory disturbance, and aphasia. The variables were reported in a dichotomized manner as “0”—no problem and “1”—problem (all variables that showed the impairment in the item were merged). Additionally, poststroke urinary incontinence (PSUI) and consciousness were used as separate variables under neurological symptoms. PSUI was evaluated during clinical examination and reported as “0”—no control and “1”—control. To report consciousness, the Mini‐Mental State Examination was also used as independent variable. Results between 0 and 17 were reported as “problem” (1) and results between 18 and 24 as “no problem” (0) in mental state (Folstein, Folstein, & McHugh, 1975). Items from the Barthel Index “feeding,” “bathing,” “grooming,” “dressing,” “toilet use,” “transfers,” “mobility,” and “stairs” were used to report the limitations in activities after stroke.

### 2.1 Statistical analysis

The mortality rates of the study population were reported in terms of prevalence and incidence. Characteristics of the study population were reported, and these characteristics compared between persons still alive and those who were deceased. Cross‐tabulation was used for describing differences in frequencies of categorical independent variables. Independent‐sample *t* tests were used to compare variables that were continuous. Descriptive statistics on the data that were not included in the analysis due to missing data on whether patients were still alive after 7 years were also conducted and compared to the total study population. The Cox proportional hazards analysis with a stepwise approach to analyze mortality was used. Three separate analyses were conducted for related pre‐stroke risk factors, neurological symptoms, and limitations of activities after stroke as independent variables, respectively. The stepwise approach was used for each analysis in the following way (Bursac, Gauss, Williams, & Hosmer, 2008): Step 1: Candidates for multiple regression were selected by running univariate regression analysis for each independent variable. If the *p*‐value for the variable in the analysis was <0.25, the factor was selected for inclusion in the analysis of Step 2; Step 2: The multiple Cox regression analysis with variables from Step 1 was conducted; Step 3: The multiple Cox regression analysis with variables with *p* < 0.25 in Step 2 was conducted; Step 4: if the model still contained variables with *p* > 0.05, repeated regression analysis was conducted by excluding this variable from analysis. The models were compared using a likelihood ratio test; Step 5: The variables that were rejected in Step 1 were reinserted one at the time. The likelihood ratio test was repeated. The model that explained the mortality best was used as a final model (Kleinbaum, Kupper, Nizam, & Rosenberg, 2013). Odds ratio and 95% confidence intervals were reported for each variable included in the final model, if the *p* value was <0.05. Statistical analyses were performed with SPSS version 20. All reported *p*‐values are two‐sided. The cumulative mortality rate was analyzed using Kaplan–Meier analysis for significant factors in each analysis to illustrate the difference in mortality.

## 3 RESULTS

The overall cumulative mortality was 24.2% within 1 year and 62.8% within 7 years. The mortality rates of the study population are reported in Table 1. The most common causes of death were cerebrovascular diseases (37%) and cancer (20%). Out of 251 patients that were included in the study, 20 (8.0%) patients were excluded due to being out of reach. Data from 231 patients were analyzed. The majority of the population were male (123 (53.2%)), age ranging from 19 to 92 years. The data analysis showed that characteristics were not significantly different between patients that were included in the final study sample and those who were not, except for smoking (*p* = 0.04) and aphasia (*p* = 0.05). Descriptive statistics for the overall study, persons still alive, and those who were deceased is shown in Table 2. Thirty‐three out of 86 persons who survived were using medication for secondary stroke prevention.

**Table 1:** Poststroke mortality within 7 years

	*N* of patients (% of all study population)	Cumulative mortality, %	Incidence	Incidence of mortality per 100,000 person‐years
Deceased				
1 month	21 (8.4%)	9.1	83	
1 year	56 (22.3%)	24.2	223	223
5 years	129 (51.4%)	55.8	514	102
7 years	145 (57.8%)	62.8	577	82
Alive	86 (34.3%)	100		
All included in the study	231 (92%)			
Excluded	20 (8%)			
All patients	251 (100%)			

**Table 2:** Descriptive statistics of stroke risk factors, neurological symptoms, and limitations of activities for the study population

Factors	Overall study population (*n* = 231), *n* (%)	Alive (*n* = 88), *n* (%)	Deceased (*n* = 143), *n* (%)	*p* a
Risk factors				
Type of stroke				
CI	185 (80)	69 (80)	116 (80)	
SICH	46 (20)	17 (20)	29 (20)	
Arterial hypertension	200 (87)	71 (83)	129 (89)	
Diabetes mellitus	49 (21)	14 (16)	35 (24)	
Atrial fibrillation	138 (60)	51 (59)	87 (60)	
Smoking	65 (28)	25 (29)	40 (28)	
Alcohol abuse	29 (13)	6 (7)	23 (16)	
Obesity	70 (30)	29 (34)	41 (28)	
Recurrent stroke	29 (13)	10 (12)	19 (13)	
Age				
Mean (max–min)	68.7 (19–52)	67.1 (36–91)	69.7 (19–92)	
Median (IQR)	71 (61–78)	68 (55.25–74)	72.0 (65–79)	
Gender				
Female	108 (47)	39 (45)	69 (47.6)	
Male	123 (53)	47 (55)	76 (52)	
Neurological symptoms				
Motor deficit	221 (96)	80 (93)	141 (97)	
Sensory disturbance	169 (73)	59 (69)	110 (76)	
Aphasia	79 (34)	20 (23)	59 (41)	0.01
Poststroke urinary incontinence	184 (80)	61 (71)	123 (85)	<0.001
Mental state	106 (46)	40 (46)	66 (46)	0.02
Limitations of activities				
Feeding				
Unable	70 (30)	10 (11)	60 (42)	<0.001
Needs help	119 (52)	55 (62)	64 (45)	
Independent	42 (18)	23 (26)	19 (13)	
Bathing				
Dependent	200 (87)	70 (79)	130 (91)	0.02
Independent	31 (13)	5 (18)	13 (9)	
Grooming				
Needs help	183 (79)	58 (66)	125 (87)	<0.001
Independent	48 (21)	30 (34)	18 (13)	
Dressing				
Dependent	97 (42)	15 (17)	82 (57)	<0.001
Needs help	105 (46)	55 (62)	50 (35)	
Independent	29 (12)	18 (20)	11 (8)	
Toilet use				
Dependent	103 (45)	20 (23)	83 (58)	<0.001
Needs help	105 (45)	53 (60)	18 (13)	
Independent	23 (10)	15 (17)	8 (6)	
Transfers				
Unable	83 (36)	14 (16)	69 (48)	<0.001
Major help	70 (30)	27 (31)	43 (30)	
Minor help	59 (26)	33 (37)	26 (18)	
Independent	19 (8)	14 (16)	5 (3)	
Mobility				
Immobile	87 (38)	16 (18)	71 (50)	<0.001
Wheelchair independent	78 (34)	34 (39)	44 (31)	
Walks with help	53 (23)	28 (32)	25 (17)	
Independent	13 (5)	10 (11)	3 (2)	
Stairs				
Unable	167 (72)	50 (57)	117 (82)	<0.001
Needs help	61 (26)	35 (40)	26 (18)	
Independent	3 (2)	3 (3)	0 (0.0)	

CI: cerebral infarction; IQR: interquartile range; SICH: spontaneous intracerebral hemorrhage.

*n* = 231.

a Only *p*‐values <0.05 have been reported.

**Figure 1:**
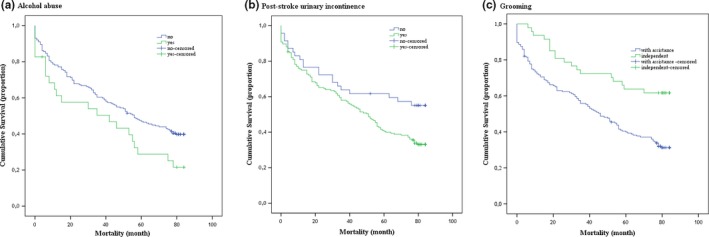
The cumulative survival curves for significant factors influencing mortality: alcohol abuse (a), poststroke urinary incontinence (b), and grooming (c)

Results from the stepwise Cox proportional hazards analysis for pre‐stroke risk factors, neurological symptoms, and limitations of activities after stroke are shown in Tables 3, 4, 5, respectively. The final model for each group of factors included only one of the factors used for the analysis. Patients who had alcohol abuse were 40% more likely to die early. The risk for those with PSUI is 1.72 times higher than those without PSUI. Independence in grooming showed a 39% lower risk of an early death. Cumulative mortality rates for alcohol abuse, PSUI, and independence in grooming have been shown in Figure 1.

**Table 3:** The results of the Cox proportional hazards analysis for pre‐stroke risk factors

	Step I	Step II	Step IIIA	Step IIIB	Step IV	Final model		
	*p*	*p*	*p*	*p*	*p*	HR	95% CI	
Arterial hypertension	0.21*	0.24			0.16			
Diabetes mellitus	0.08*	0.16*	0.10		0.09			
Atrial fibrillation	0.97				0.86			
Smoking	0.75				0.50			
Alcohol abuse	0.05*	0.05*	0.06*	0.05*	0.05	0.60	0.38	0.95
Obesity	0.31			0.17			
Recurrent stroke	0.82			0.95			
Age	0.28			0.16			
Gender	0.63				0.42			
Likelihood ratio test, *p*		>0.20						
			>0.10					

95%CI: 95% confidence interval; HR: hazard ratio.

*Variables included in the next step of the analysis.

**Table 4:** The results of Cox proportional hazards analysis for neurological symptoms after stroke as explanatory factors

	Step I	Step II	Step IIIA	Step IIIB	Step IV	Final model		
	*p*	*p*	*p*	*p*	*p*	HR	95% CI	
Paresis	0.17*	0.34			0.17			
Sensory disturbance	0.40				0.33			
Aphasia	0.06*	0.17*	0.19		0.19			
Mental status (MMSE)	0.94				0.76			
PSUI	0.02*	0.10*	0.05*	0.02*	0.02	1.71	1.08	2.72
Likelihood ratio test, *p*		*p* > 0.20						
			*p* > 0.10					

95%CI: 95% confidence interval; HR: hazard ratio; PSUI: poststroke urinary incontinence.

*Variables included in the next step of the analysis.

**Table 5:** The results of Cox proportional hazards analysis for limitations of activities after stroke as explanatory factors

	Step I	Step II	Step IIIA	Step IIIB	Step IV	Final model		
	*p*	*p*	*p*	*p*	*p*	HR	95% CI	
Feeding	0.02*	0.98			0.84			
Bathing	0.03*	0.48			0.75			
Grooming	<0.005*	0.01*	<0.001*	<0.005	<0.005	0.39	0.24	0.65
Dressing	0.02*	0.54			0.46			
Toilet use	0.04*	0.28			0.97			
Transfers	0.01*	0.63			0.15			
Mobility	0.02*	0.22*	0.18		0.18			
Stairs	0.25				0.10			
			*p *= <0.10					

95%CI: 95% confidence interval; HR: hazard ratio.

*Variables included in the next step of the analysis.

## 4 DISCUSSION

The results of this study show the important factors that can influence long‐term mortality after stroke for patients that have been treated in a stroke unit. Alcohol abuse as a pre‐stroke risk factor, poststroke urinary incontinence as a neurological symptom, and dependence in grooming as a factor of disability were associated with earlier mortality in the first 7 years after stroke.

Within the study population, every tenth person died within the first month after event. One in four persons died within the first year, every second within five years, and only one third of all patients survived more than 7 years after stroke. Compared to other similar studies, the mortality rate was at least two times higher in this stroke population from Latvia (Chang, Lee, Tseng, & Huang, 2010). Although the incidence of mortality has halved since 1995 (London KsC, 2017), mortality rates have been almost three times higher than in Nordic countries (Wilkins et al., 2017). One possible explanation of these results could be that as a consequence of the financial crisis of 2008, healthcare services funding in Latvia decreased. The governmental funding of healthcare in Latvia remains at 2.9% of GDP, regardless of the time that has passed since the crisis. This is half as much as in other Baltic and Northern Europe countries (London KsC, 2017). Moreover, along with decreased funding from the government, patient fees were increased. This could lead to dropouts from the continuity of adequate stroke care and consequently an increased mortality. There are several worrying facts that raise from the results of this study. Firstly, mortality rates at discharge from this stroke unit in Latvia are somewhat similar to mortality rates in other European countries. Then, within 5 years, these rates differ considerably. Secondly, more than one third of deaths in this study population occurred due to stroke, and one third of those who died, died due to a repeat stroke. Thirdly, fewer than half of those who survived 7 years after stroke used medication for secondary stroke prophylaxis. This all leads to the belief that there are severe disadvantages in the care of stroke patients that follow acute treatment in Latvia, such as secondary stroke prevention and continuity of care.

Use of alcohol before stroke turned out to be the risk factor that is most strongly associated with an earlier death after stroke, far ahead of such well‐studied risk factors as diabetes, atrial fibrillation, smoking (Kammersgaard & Olsen, 2006), or age (Koton, Tanne, Green, & Bornstein, 2010). These findings contradict overall knowledge of the most important risk factors. However, the single‐factor analysis showed arterial hypertension, diabetes, and older age were also important risk factors. Alcohol as a risk factor for mortality is infrequently mentioned in the literature. However, there is some evidence that excessive usage of alcohol is a risk factor for hemorrhagic stroke (Qureshi et al., 2001) and cerebral infarction (Sacco et al., 2006). The reasons for those well‐known risk factors not showing themselves as important issues in long‐term prognosis in this study could be connected to the fact that occurrence of them in both groups (dead and alive) was high and did not differ significantly, due to the rather small study population. However, the proportion of those who reported themselves being excessive alcohol users was considerably smaller and 23 patients out of 29 died within the study period.

The results of the analysis of neurological symptoms early after stroke as indicators of mortality showed that there were three individual risk factors—paresis, aphasia, and PSUI—that could have some level of influence on the outcome. The multiple factor analysis showed urinary incontinence as the most dominant factor. In 1988, Fullerton published a study that showed 21 factors that can impact the result of stroke in the first 48 hr after stroke, in terms of the chance of death and disability in the first 6 months. Among the most important were such factors as perception, strength of muscles in upper and lower limbs, and disturbance of consciousness, but not incontinence (Fullerton, Mackenzie, & Stout, 1988). Nowadays, there is evidence that PSUI has a predictive value regarding higher risk of mortality, worse prognosis of recovery, longer time spent in hospital, and more frequent hospital visits (John, Bardini, Megevand, Combescure, & Dallenbach, 2016; Nakayama, Jorgensen, Pedersen, Raaschou, & Olsen, 1997). Urinary incontinence is an “involuntary loss of urine, which is objectively demonstrable and social or hygienic problem.”(Abrams, Blaivas, Stanton, & Andersen, 1988). It is a multifactorial problem with different directions of causalities. It can manifest as a result of the following mechanisms: as a consequence of disturbed urination function as classified in the Body Function section of the International Classification of Functioning, Disability, and Health (ICF); functional urinary incontinence as a secondary outcome of impairments of other body functions (such as immobility, aphasia, cognitive disturbance, or depression) or due to urinary infections caused by the placement of indwelling urinary catheter (iatrogenic urinary incontinence). The problem with the last cause is that in daily practice clinicians tend to focus more on other neurological symptoms without giving the needed attention to bladder dysfunction. Very commonly, the indwelling urinary catheter is placed when a patient is admitted to a stroke unit with an unstable medical condition. This catheter is often removed, not at the same time as the medical condition stabilizing, but later. Therefore, the recognition and proper care of the problem often is delayed. Education of hospital staff in the early recognition, assessment, and proper management of the problems mentioned above could contribute to a better outcome. Moreover, proper documentation of urinary incontinence is an issue that also has to be addressed (Watson, Brink, Zimmer, & Mayer, 2003).

The third analysis of this study was done on the limitations of activities early after stroke as predictive factors on long‐term mortality. These factors were analyzed separately from neurological factors due to the absence of direct causality between them. Moreover, the technologies used in rehabilitation aim at improving independence in activities of the person, by using adaptive or compensatory techniques, among others. Therefore, early recognition of limitations that can be targeted in the process of rehabilitation is important for rehabilitation professionals.

Almost all factors that were included in the analysis of this study showed statistical significance as single factors. Patients with more limitations in activities were more prone to die earlier. The approach of analyzing the activity limitation as separate items that was used in this study is rather unique, since most research has been focused on the evaluation of neurological symptoms, general disability, or level of dependence.

The final model of stepwise regression analysis showed that the most dominant predictor of dying earlier in this group was grooming. This is also an interesting finding, considering that usually grooming is not recognized as the primary issue among the other dimensions of disability. In the acute phase of stroke, rehabilitation interventions are usually focused on activities such as mobility and eating. This focus can be explained by the necessity to prevent complications and the well‐defined nature of these activities related to mobility and eating. However, grooming requires more complex skills that include mouth hygiene, brushing hair, nail cutting, and shaving.

### 4.1 Strengths and limitations

This study includes mortality data on patients who have been discharged from only one stroke unit within a six‐month period, forming a small proportion of the overall stroke population in Latvia. However, in 2009 this was the only stroke unit in Latvia. It has been estimated that currently approximately 60% of those who suffer from a stroke are admitted to one of seven stroke units. Therefore, this study does not provide the true picture of mortality from stroke in Latvia. Introduction of a national quality register for stroke in Latvia would help to collect representative data on mortality and care of stroke patients. Moreover, different organization of stroke care, including available medical and rehabilitation staff and used technologies, can determine different outcomes. It means that data cannot be generalized to national level. On the other hand, research patients were evaluated when coming into the stroke unit by using standardized measures and were under observation for a 7‐year period, which can be considered as an advantage of this study. A relatively small group of patients (8%) were excluded from analysis due to being out of reach; therefore, there is no reason to believe that it has affected the results. Another limitation of this study is that it was not possible to gather reliable data on causes of mortality that would be important for additional information on the problems related to secondary prevention after stroke. There is also limited information on discharge destination; therefore, it cannot be used to report statistically reliable data. However, existing data and clinical experience of the authors suggest that vast majority of patients has returned home and assistance is provided by relatives. This is due to cultural peculiarities and organization of social care services in Latvia.

## 5 CONCLUSIONS

Alcohol abuse as a pre‐stroke risk factor, poststroke urinary incontinence as a neurological symptom, and dependence in grooming as an activity limitation shortly after stroke are associated with poststroke mortality during the first 7 years after stroke for persons that have been treated in a Latvian stroke unit.

## CONFLICT OF INTEREST

The authors declare that they have no financial or non‐financial competing interests.
